# Expression of plasminogen activators in preimplantation rat embryos developed *in vivo *and *in vitro*

**DOI:** 10.1186/1477-7827-3-7

**Published:** 2005-02-10

**Authors:** Eliahu D Aflalo, Uriel A Sod-Moriah, Gad Potashnik, Iris Har-Vardi

**Affiliations:** 1 Department of Life Sciences, Ben-Gurion University of the Negev, P.O.Box 653, Beer-Sheva 84105, Israel; 2 Fertility and In vitro Fertilization (IVF) Unit, Department of Obstetrics and Gynecology, Soroka University Medical Center, Faculty of Health Sciences, Ben-Gurion University of the Negev, Israel

## Abstract

**Background:**

Embryo implantation plays a major role in embryogenesis and the outcome of pregnancy. Plasminogen activators (PAs) have been implicated in mammalian fertilization, early stages of development and embryo implantation. The invasion of trophoblast cells into the endometrium during the implantation process can be blocked by inhibitors of serine proteases, illustrating the role of these enzymes in the invasion process. As in vitro developing embryos resulted in lower implantation rate than those developed in vivo we assume that a reduced PAs activity may lead to it. There is hardly any information regarding qualitative or quantitative differences in expression of PAs in preimplantation embryos, or comparisons between in vivo and in vitro developed embryos. The purpose of this study was to assess the expression of urokinase type (uPA) and tissue type (tPA) plasminogen activators in in vivo and in vitro preimplantation development in rat embryos using immunofluorescence confocal microscopy and computerized image analysis.

**Methods:**

Zygotes, 2-cell, 4-cell, 8-cell, morula and blastocyst stages of development were flushed from the reproductive tract (control groups) of Wistar rats. Zygotes were flushed and grown in vitro to the above mentioned developmental stages and comprised the experimental groups. Immunofluorescence microscopy and computerized image analysis were used to evaluate both qualitative (localization) and quantitative expression of plasminogen activators.

**Results:**

uPA and tPA were found to be expressed in rat embryos throughout their preimplantation development, both in vivo and in vitro. While uPA was localized mainly in the cell cytoplasm, the tPA was detected mainly on cell surface and in the perivitelline space. In blastocysts, both in vivo and in vitro, uPA and tPA were localized in the trophectoderm cells. Total uPA content per embryo was higher in the in vivo as compared with the in vitro developed embryos at all stages measured. Blastocyst uPA content was significantly low as compared with the four-cell, eight-cell, and morula stages. Total tPA content was higher in embryos developed in vivo than those developed in vitro except for the 4-cell and 8-cell stages.

**Conclusion:**

In vitro embryo development leads to lower PAs expression in a stage dependent manner as compared with in vivo developing controls. The enzymes studied vary probably in the ratio of their active and inactive forms as there is no correlation between their content and the activity observed in our previous study. The localization of both PAs in the blastocysts' trophectoderm supports the assumption that PAs plays a role in the implantation process in rats.

## Background

Plasminogen activators (PAs) and matrix metalloproteinases (MMPs) have been implicated in mammalian gametogenesis [1], ovulation [2,3], fertilization [4,5], early stages of development and embryo implantation [6,7]. The PAs are serine proteases, which convert the inactive plasminogen to the potent protease plasmin. Plasmin can degrade directly or indirectly, through the activation of metalloproteinase zymogens, all components of the extracellular matrix [8,9]. There are two types of PAs, tissue-type plasminogen activator (tPA) and urokinase-type plasminogen activator (uPA). Plasminogen, its activators and inhibitors, participate in the implantation process. Trophoblast cells of human blastocysts cultured in vitro produced PAs during the period corresponding to the in vivo invasion into the endometrium [10]. In embryos of the homozygous t^w73 ^mouse mutant, PAs were reduced and was concomitantly associated with implantation failure [11]. The invasion of trophoblast cells during the implantation process could be blocked by inhibitors of serine proteases, illustrating the role of these enzymes in the invasion process [12,13]. In the human, embryo implantation following in vitro fertilization and embryo transfer (IVF-ET) is considered to play a major role in the success of the treatment. Only 12% of the transferred embryos are able to successfully implant [14].

In the implantation process, two major factors participate: the uterus undergoes changes that prepare it for the arrival and implantation of embryos, and the embryos undergo cellular reorganization that enables them to penetrate the endometrium and to form the placenta. We assume that one of the reasons for low implantation rate of embryos developed in vitro involves reduced PAs activity.

In a previous study we demonstrated differences in PAs activities between in vivo and in vitro preimplantation developed embryos. In both, uPA activity increased from the zygote towards the blastocyst stage while tPA activity remained relatively unchanged. However, tPA and uPA activities were lower in in vitro developed embryos as compared with in vivo developing ones, at all developmental stages, which may lead to a reduced implantation rate of in vitro developed embryos [15].

There is hardly any information regarding qualitative or quantitative differences in expression of PAs in preimplantation embryos, or comparisons between in vivo and in vitro developed embryos. Therefore, the purpose of this study was to investigate the PAs expression and localization during embryo development in vivo and in vitro by immunofluorescence confocal microscopy.

## Methods

The following study was approved by the Institutional committee for animal care and ethics at Ben-Gurion University of the Negev, Beer-Sheva, Israel.

### Animals

Mature female Wistar rats 2–3 months old, weighing 180–230 g were used. The animals were kept in a temperature-controlled room maintained at 22–24°C with lighting regimen of 14 hours light 10 hours dark (light on 5:00 AM – 7:00 PM). The rats were allowed free access to rat chow and tap water.

Daily vaginal smears were taken at 10:00 AM, and the stage of estrous cycle was determined. Overnight caging of a proestrous female with a male of proven fertility induced pregnancy. The next day, the presence of a vaginal plug or spermatozoa in the vaginal smear was designated as day 1 of pregnancy.

### Collection of embryos

Zygotes, two-cell, four-cell, eight-cell embryos and morulae were flushed with rat 1-cell embryo culture medium (R1ECM) [16] from oviducts at days 1, 2, 3 and 4 of pregnancy, respectively, and blastocysts at day 5 from the uterine horns. All equipment and media used were sterile. Ovary-oviduct complexes were removed from anesthetized animals. The complexes were placed in R1ECM, and the oviducts were separated under a dissecting microscope. 30-gauge blunt-end needle attached to a syringe containing R1ECM was inserted through the oviductal end held by forceps surrounding the needle and tube. Embryos were gently flushed into 35-mm-diameter culture dish. Embryos were washed 3 times by transfer into fresh R1ECM to remove cell debris and any maternal factors present in the oviduct.

Zygotes in their cumulus mass were flushed and the cumulus cells were removed by gentle aspiration through a micropipette (diameter, 150–200 μm) several times in R1ECM containing 80 U/mL of hyaluronidase. Clean zygotes were washed 3 times by transfer into fresh R1ECM to remove traces of hyaluronidase. Flushed embryos were collected with a mouth-controlled micropipette (diameter, 150–200 μm).

Blastocysts were flushed from the uterine horns by insertion of a 23-gauge needle attached to a syringe containing R1ECM. Flushed, free-floating blastocysts were collected into a polypropylene tube inserted through the vagina and pushed gently to surround the cervical openings. Tubes were removed, and their contents were poured into Petri dishes. Blastocysts were washed and collected as described for zygotes and embryos. These embryos developed in vivo were the control groups.

### Embryo culture

As described for the in vivo embryos, clean zygotes were grown in vitro to the same developmental stages as controls; these were the experimental groups. Each group of embryos consisted of 25–35 embryos collected from six pregnant females. This was repeated three times for each developmental stage (total of about 90 embryos per stage). Groups of 25–35 zygotes were placed into 35-mm-diameter culture dishes (Nunc, Roskilde, Denmark) containing 50μL of R1ECM medium under a layer of mineral oil (previously equilibrated to the experimental conditions) and cultured at 37°C under 5% CO_2 _in air. This medium was shown by Miyoshi et al. [16] to enable rat embryo culture to the blastocyst stage. In a comparison of various media at our laboratory, R1ECM was found to be the best medium to enable a synchronous development of embryos (95% of total) to the blastocyst stage. The developing embryos seemed to be normal in their morphology, with almost no fragmentation. At the end of incubation, embryos were washed 3 times with fresh R1ECM.

### Embryo immunocytochemistry

The method used was basically that of Dubey et al. [17] with various modifications. Groups of 20–25 embryos at different developmental stages from the experimental and control groups were fixed in 4% paraformaldehyde in phosphate buffered saline (PBS) at room temperature and washed twice in PBS, PH 7.4 for 5 minutes. Five percent bovine serum albumin (BSA) in PBS was used for dilution of antibodies and washings (PBS-BSA). The embryos were washed four times in PBS-BSA before immunoreaction. Embryos, randomly chosen were either exposed to polyclonal rabbit anti rodent uPA or rabbit anti rat tPA (American Diagnostics, Pendelton, IN) at a concentration of 4μg/mL. Embryos were then incubated overnight in 50μL of each antibody solution under paraffin oil in a 35 mm culture plate in a moist chamber at 4°C. The embryos were then washed four times in PBS-BSA and incubated with Cy3-conjugated goat anti-rabbit IgG (Jackson ImmunoResearch Laboratories, Inc., West Grove, PA) at 37°C for 60 minutes. The conjugated antibody was used at a dilution of 1:300 in PBS-BSA. After incubation with the secondary antibody, the embryos were washed again in PBS-BSA and stained with DNA stain 4', 6-diamidino-2-phenylindole (DAPI) (Vector Laboratories, Burlingame, CA) and mounted in Flouromount – G (Southern Biotechnology Associates, Inc. Birmingham, AL) to minimize quenching. To confirm that the fluorescence observed was neither attributable to nonspecific binding of the secondary antibody nor to formaldehyde-induced autofluorescence, negative controls (without primary antibody) were established during each immunoreaction procedure. The immunocytochemistry staining procedure was repeated three times for each stage of embryo development on different batches of embryos.

### Image Analysis

The distribution and concentration of PAs in the embryos were visualized by fluorescent microscopy on a Zeiss laser scanning confocal microscope equipped with an X100 objective. Z-sections and XZ-sections were obtained from 3D scanning by using LSM510 software (Zeiss, Feldbach, Switzerland) The PAs density for each embryo was computed by image analysis based on the same principles as manual counting described elsewhere [17]. The embryos' fluorescent images were downloaded using an image analysis software, ImagJ (NIH, Bethesda, MD). These images were stored in the computer by use of pixels. All the slices obtained from 3D scanning were of 0.7μ and were analyzed by counting the number of pixels of the Cy3 (red color) in the whole slice. Total pixels in a whole embryo were calculated by summing the number of pixels in all the slices of an embryo. This method showed the total amount of PAs expression in each embryo. The number of pixels in an embryo represents the intensity of PAs staining (fluorescence), which is in turn proportional to the amount of PAs in that embryo. Each experimental group consisted of 8–10 embryos and the measurements repeated three times with different batches of embryos from each developmental stage stained at different times (total number of 24–30 embryos per stage).

### Statistical analysis

Data are expressed as means ± SEM. Statistical analysis was performed with two-way analysis of variance, followed by the least significant differences test for multiple comparisons using computer software (Statistica 6.0, Statsoft, Inc. Tulsa, OK). P < 0.05 was defined as statistically significant difference.

## Results

### PAs localization

**Figure 1:**
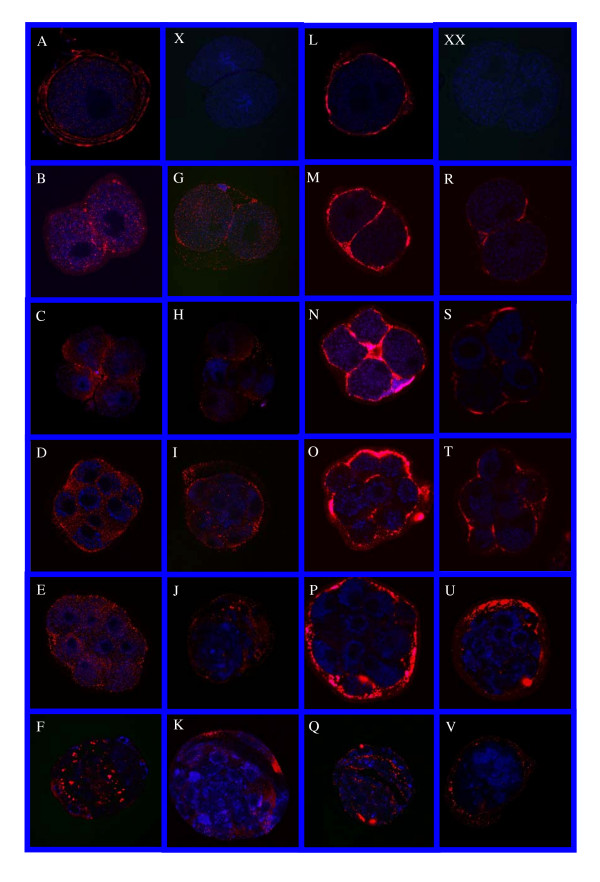
**PAs localization. **Expression of uPA (A-K) and tPA (L-V) in preimplantation developing rat embryo stages grown in vivo and in vitro. (A, L)-Zygote. (B, G, M, R)-2-cells. (C, H, N, S)-4-cells. (D, I, O, T)-8-cells. (E, J, P, U)-Morula. (F, K, Q, V)-Blastocyst. (A-F)-uPA in vivo. (G-K)-uPA in vitro. (L-Q)-tPA in vivo. (R-V)-tPA in vitro. (X)-2-cell uPA negative control. (XX)- 2-cell tPA negative control.

Immunohistochemical staining for the location of tPA and uPA in preimplantation embryos developed in vivo and in vitro are shown in figure 1. The PAs were detected in all stages of embryo development, both in in vivo and in vitro (Fig. 1A–V). The uPA was expressed in the cell cytoplasm and plasma membrane (Fig. 1A–K) while tPA was detected on the cell membrane and in the perivitelline space (Fig. 1L–V). In blastocysts developed in vivo and in vitro PAs were localized mainly in the trophectoderm (Fig. 1F, K, Q, V). There was no difference in PAs localization comparing in vivo and in vitro developed embryos at the same stage.

### Quantitative measurement of uPA

**Figure 2:**
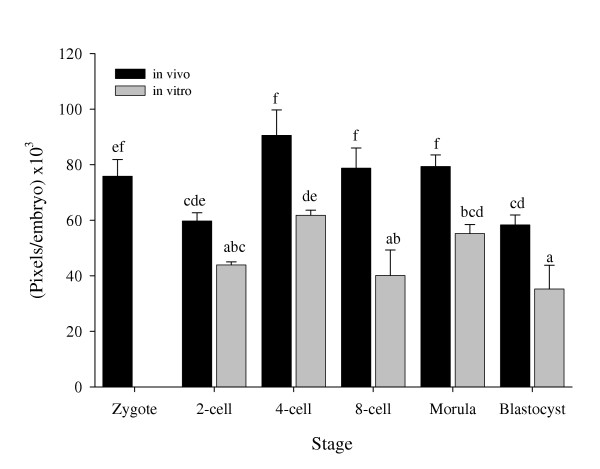
**Quantitative measurement of uPA. **Quantitative (Pixels/Embryo) uPA expression in preimplantation rat embryos developed in vivo and in vitro. Different letters represent statistically significant differences (P < 0.05).

Quantitative measurement of total uPA in an embryo at each stage showed significantly lower expression (p < 0.01) in in vitro developed embryos from the 4-cell stage up to the blastocyst stage compared with the in vivo developed corresponding timepoint. The highest expression of uPA was found in in vivo developed embryos from 4-cell to the morula stage (90.58, 78.78 and 79.35 Pixels per embryo × 10^3^, respectively, Fig. 2). In the in vitro developed embryos, a significant increase (p < 0.01) in uPA expression was found from the 2-cell stage to the 4-cell stage (43.91 and 61.86 Pixels per embryo × 10^3^, respectively).

### Quantitative measurement of tPA

**Figure 3:**
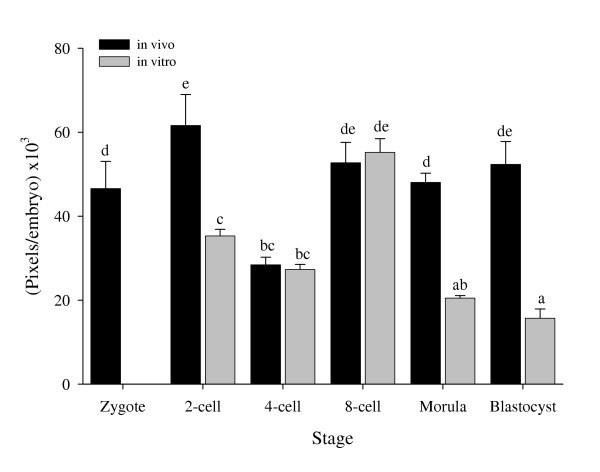
**Quantitative measurement of tPA. **Quantitative (Pixels/Embryo) tPA expression in preimplantation rat embryos developed in vivo and in vitro. Different letters represent statistically significant differences (P < 0.05).

Total tPA expression in a whole embryo showed highest expression in in vivo developed embryos at the 2-cell, 8-cell, morula and blastocyst stages (61.59, 52.71, 48.03 and 52.34 Pixels per embryo × 10^3^, respectively, Fig. 3). At the 2-cell stage, morula and blastocyst, a significantly lower expression (p < 0.01) was found in in vitro developed embryos as compared with the in vivo ones.

## Discussion

Our study demonstrates that uPA and tPA are expressed throughout all stages of preimplantation development of rat embryos. Zhang et al. [18] reported expression of uPA gene and uPA activity in preimplantation rat embryos developed in vitro and Khamsi et al. [19] reported the presence of mRNA for uPA in human blastocysts. However, information about the expression and immunolocalization of uPA and tPA has not been reported in in vivo nor in in vitro preimplantation developing rat embryos. We present here a quantitative measurement of PAs expression in a whole embryo allowing comparison of embryos at different developmental stages, grown in vivo or in vitro.

The results show localization of immunoreactive uPA in the embryonic cell cytoplasm and plasma membrane in all developmental stages both in vivo and in vitro, while tPA is detected on the cell membrane and in the perivitelline space. In the blastocyst stage, PAs are localized mainly in the trophectoderm. In our previous study [15] we showed that the activity of uPA was higher than that of tPA in the blastocyst. Its presence in the trophectoderm combined with its high activity in this stage, support the assumption that uPA is important for proper implantation. This assumption is supported by the study of Kubo et al. [20] who showed that inhibition of PAs activity prevents the adhesion of mouse embryos to decidual cells grown in vitro. In addition, trophoblast cells grown in vitro showed PAs activity at the time of their penetration into the endometrium in vivo and uPA was the major enzyme secreted from trophectoderm cells with the highest activity on days five to seven of pregnancy [7]. The exact source(s) of the immunoreactive PAs in in vivo developing embryos cannot be identified. The serum, oviduct and endometrium could be contributing sources as suggested in previous studies [10,21-23]. Lack of the these source(s) in in vitro situation may lead to lower implantation ability of embryos as shown earlier [15].

Pro-uPA is synthesized as an inactive single chain that can be stored or secreted. The secreted pro-uPA can be cleaved to produce the two-chain active molecule, uPA, by the aid of limited proteolytic activity of plasmin [24]. The secreted pro-uPA or the active uPA can be found free in cytoplasm and extracellular matrix or bound to a membrane uPA receptor [25]. Whether the uPA identified in this study is the inactive pro-uPA or the active uPA associated with the embryonic cell membrane uPA receptor, is unknown. In our previous work we have shown an increase in uPA activity towards the blastocyst stage in in vivo and in in vitro developing embryos [15]. The results of the present study, showing lower expression of uPA in the blastocyst stage, may suggest a shift of uPA from the inactive form to the active form resulting in an increase of activity despite the reduction in its expression.

High tPA expression was detected at the zygote stage which is in accordance with high tPA activity found in this stage [15]. This is supported by the report of Zhang et al. [18] who showed presence of tPA mRNA in rat oocytes and two-cell embryos. The embryonic genome of rats and mice start to be expressed at the 2-cell stage [26] and the high tPA levels in the zygote are probably due to maternal mRNA expressed and accumulated in the oocyte [27].

The embryonic extracellular matrix is in a continuous turnover during the embryonic development. The 8-cell stage is characterized by structural changes taking place in the embryo during the compaction process. It is therefore very likely that such changes at the 8-cell stage could be associated with increased tPA expression and activity which is known to participate in tissue remodeling [8]. The high increase in tPA expression from the 4-cell stage to the 8-cell stage in in vitro developed embryos suggests de novo synthesis of tPA since there is no extraembryonic tPA source but the embryos in the culture.

Lower expression of uPA was observed in in vitro developed embryos as compared with in vivo ones from the 4-cell up to the blastocyst stage while tPA expression was lower only in the morula and blastocyst stages. This could be explained by reduced metabolic activity in the in vitro developed embryos as suggested by Krisher et al. [28]. In addition, in vitro conditions may lead to a slower cell division rate which may result in a blastocyst comprised of fewer cells and decreased ability to hatch from the zona pellucida [28,29]. Carroll et al. [30] showed that the oviduct is also a source of PAs, which could attach to receptors on embryonic cell membrane, and this source is lacking in in vitro developing embryos. It should be noted that any culture media would lack maternal factors, known or yet unknown, which affect embryo development and thus, implantation rate, through their effect on the PA/plasmin system.

Additional studies addressing the regulation of PA/Plasmin system by adding exogenous factors may provide insights into its role in early embryo development and implantation.

## Conclusions

The purpose of the study was to determine the relative importance of tPA and uPA in preimplantation embryo development. In vitro embryo development leads to lower PAs expression in a stage dependent manner as compared with in vivo developing ones. The localization of both PAs in the blastocysts' trophectoderm supports the assumption that PAs may play a role in the implantation process in rats.

## Authors' contributions

EDA participated in the planning of the project, carried out the animal experimentation, immunohistochemistry and the image analysis studies. USM participated in the planning of the project, animal experimentation and participated in preparation of the manuscript. GP participated in preparation of the manuscript. IHV participated in the planning of the project, statistical analysis and in preparation of the manuscript.

## References

[B1] Huarte J, Belin D, Vassalli JD (1985). Plasminogen activator in mouse and rat oocyte: Induction during meiotic maturation. Cell.

[B2] Tsafriri A, Bicsak TA, Cajadder T, Ny T, Hsueh AJ (1989). Suppression of ovulation rate by antibodies to tissue-type plasminogen activator and alphe 2-antiplasmin. Endocrinology.

[B3] Strickland S, Beers WH (1976). Studies on the role of plasminogen activator in ovulation. In vitro response of granulosa cells to gonadotropins, cyclic nucleotides, and prostaglandins. Journal of Biological Chemistry.

[B4] Huarte J, Vassalli JD, Belin D, Sakkas D (1993). Involvement of plasminogen activator/plasmin proteolityc cascade in fertilization. Dev Biol.

[B5] Zhang X, Rutledge J, Khamsi F, Armstrong DT (1992). Release of tissue-type plasminogen activator by activated rat eggs and its possible role in the zona reaction. Mol Reprod Dev.

[B6] Menino JAR, Hogan A, Schultz GA, Novak S, Dixon W, Focroft GH (1997). Expression of proteinases and proteinase inhibitors during embryo-uterine contact in the pig. Development Genet.

[B7] Sappino AP, Huarte J, Belin D, Vassalli JD (1989). Plasminogen activators in tissue remodeling and invasion: mRNA localization in mouse ovaries and implanting embryos. J Cell Biol.

[B8] Dano K, Andersen PA, Grondahl-Hansen J, Kristensen P, Nilsen LS, Skriver J (1985). Plasminogen activators, tissue degradation and cancer. Adv Cancer Res.

[B9] Robbins KC, Summaria L, Hsieh B, Shah RG (1967). The peptide chain of human plasmin.  mechanism of activation of human plasminogen to plasmin. J Biol Chem.

[B10] Queenan JT, Lee-Chuan K, Arboleda CE, Ulloa-Aguirre A, Golos TG, Cines DB, Strauss III JF (1987). Regulation of urokinase- type plasminogen activator production by cultured human cytotrophoblasts. J Biol Chem.

[B11] Axelrod HR (1985). Altered trophoblast functions in implantation-defective mouse embryos. Dev Biol.

[B12] Kruithof EKO, Takada A, Shumama MM and Collen D (1990). Plasminogen activator inhibitor type 2: biochemical and biological aspects. Protease inhibitors.

[B13] Loskutoff DJ, Sawdey M, J. M (1989). Type 1 plasminogen activator. Progress in Hemostasis and Thrombosis.

[B14] Schleve LA, Wilcox LS (2002). Use of assisted reproductive technology in united states, 1996 and 1998. JAMA.

[B15] Aflalo ED, Sod-Moriah UA, Potashnik G, Har-Vardi I Differences in the implantation rate of in vivo and in vitro developed rat embryos: A possible role for plasminogen activators. Fertility and Sterility.

[B16] Miyoshi K, Abeydeera LR, Okuda K, Niwa K (1995). Effects of osmolarity and amino acids in a chemically defined medium on one-cell embryos.. J Reprod Fertil.

[B17] Dubey AK, Cruz JR, Hartog B, Gindoff PR (2001). Expression of the av integrin adhesion molecule during development of preimplantation human embryos. Fertil Steril.

[B18] Zhang X, Kidder GM, Zhang C, Khamsi F, Armstrong DT (1994). Expression of plasminogen activator genes and enzymatic activities in rat preimplantation embryos. J Reprod Fertil.

[B19] Khamsi F, Armstrong TD, Zhang X (1996). Expression of urokinase-type plasminogen activator in human preimplantation embryos. Mol Hum Reprod.

[B20] Kubo H, Spindle A, Pedersen RA (1981). Inhibition of mouse blastocyst attachment and outgrowth by protease inhibitors. J Exp Zool.

[B21] Martin O, Arias F (1982). Plasminogen activator production by trophoblast cells in vitro: effect of steroid hormones and protein synthesis inhibitors. Am J Obstet Gynecol.

[B22] Casslen B, Andersson A, Nilsson IM, Astedt B (1986). Hormonal regulation of the release of plasminogen activators and of a specific activator inhibitor from endometrial tissue in culture. Proceedings of the Society for Experimental Biology and Medicine.

[B23] Huber K, Kirchheimer J, Binder BR (1984). Characterization of a specific anti-human urokinase antibody: development of a sensitive competition radioimmunoassay for urokinase antigen. J Lab Clin Med.

[B24] Ploug M, Behrendt N, Lober D, Dano K (1991). Protein structure and membrane anchorage of the cellular receptor for urokinase-type plasminogen activator. Seminars in Thrombosis and Hemostasis.

[B25] Ellis V, Behrendt N, Dano K (1991). Plasminogen activation by receptor-bound urokinase. A kinetic study with both cell-associated and isolated receptor. J Biol Chem.

[B26] Telford NA, Watson AJ, Schultz GA (1990). Transition from maternal to embryonic control in early mammalian development: A comparison of several species. Molecular Reproduction and Development.

[B27] Huarte J, Belin D, Vassalli A, Strickland S, Vassalli JD (1987). Meiotic maturation of mouse oocytes triggers the translation and polyadenylation of dormant tissue-type plasminogen activator mRNA. Genes and Development.

[B28] Krisher RL, Lane M, Bavister BD (1999). Developmental competence and metabolism of bovine embryos cultured in semi-defined and defined culture media. Biol Reprod.

[B29] Bowman P, McLaren A (1970). Cleavage rate of mouse embryos in vivo and in vitro. J Embryol Exp Morphol.

[B30] Carroll PM, Richards WG, Darrow AL, Wells JM, Strickland S (1993). Preimplantation mouse embryos express a cell surface receptor for tissue-plasminogen activator. Dev.

